# Gas Plasma Exposure Alters Microcirculation and Inflammation during Wound Healing in a Diabetic Mouse Model

**DOI:** 10.3390/antiox13010068

**Published:** 2024-01-02

**Authors:** Anke Schmidt, Debora Singer, Henrike Aden, Thomas von Woedtke, Sander Bekeschus

**Affiliations:** 1 ZIK *plasmatis*, Leibniz Institute for Plasma Science and Technology (INP), Felix-Hausdorff-Str. 2, 17489 Greifswald, Germany; 2 Clinic and Policlinic for Dermatology and Venerology, Rostock University Medical Center, Strempelstr. 13, 18057 Rostock, Germany; 3 Institute of Hygiene and Environmental Medicine, Greifswald University Medical Center, Sauerbruchstr., 17475 Greifswald, Germany

**Keywords:** angiogenesis, diabetes mellitus, hyperspectral imaging, plasma medicine, reactive species, ROS, RNS, redox regulation

## Abstract

Diabetes can disrupt physiological wound healing, caused by decreased levels or impaired activity of angiogenic factors. This can contribute to chronic inflammation, poor formation of new blood vessels, and delayed re-epithelialization. The present study describes the preclinical application of medical gas plasma to treat a dermal, full-thickness ear wound in streptozotocin (STZ)-induced diabetic mice. Gas plasma-mediated effects occurred in both sexes but with gender-specific differences. Hyperspectral imaging demonstrated gas plasma therapy changing microcirculatory parameters, particularly oxygen saturation levels during wound healing, presumably due to the gas plasma’s tissue delivery of reactive species and other bioactive components. In addition, gas plasma treatment significantly affected cell adhesion by regulating focal adhesion kinase and vinculin, which is important in maintaining skin barrier function by regulating syndecan expression and increasing re-epithelialization. An anticipated stimulation of blood vessel formation was detected via transcriptional and translational increase of angiogenic factors in gas plasma-exposed wound tissue. Moreover, gas plasma treatment significantly affected inflammation by modulating systemic growth factors and cytokine levels. The presented findings may help explain the mode of action of successful clinical plasma therapy of wounds of diabetic patients.

## 1. Introduction

The skin represents a multifunctional complex barrier against pathogens and functions as natural defense of the body. Wounds disrupt the integrity and function of the skin. However, physiological wound healing involves a highly organized and coordinated sequence of events involving various cellular and molecular mechanisms [1]. In the initial phase of wound healing, inflammation occurs as a response to tissue injury. It involves recruiting immune cells, releasing inflammatory mediators, and debris clearance. During the proliferation and remodeling phase, new blood vessels are formed (angiogenesis), and fibroblasts produce collagen to rebuild the extracellular matrix. Epithelial cells migrate to close the wound [2]. Patients with diabetes mellitus (DM) are particularly prone to develop chronic wounds, i.e., wounds that fail to heal within 3–6 weeks post-infliction. Etiologically, chronic wounds are primarily diabetic foot ulcers, venous ulcers, and pressure sores [3]. Several host-related factors impair healing, including elevated blood glucose levels, neuropathy, prolonged and exaggerated inflammation, impaired immune responses, and blood circulation [4]. The latter often results in microvascular complications, such as reduced blood flow (peripheral vascular disease) and nerve damage (neuropathy) [5]. These conditions further impede wound healing by reducing oxygen and nutrient supply to the wound region [6]. Moreover, diabetic wounds often have impaired angiogenesis, leading to delayed re-epithelialization [7]. Currently, no chronic wound model in mice resembles all features of chronic wound healing and human diabetes, although several rodent models have been established to study diabetes-delayed wound healing. Streptozotocin (STZ) is a naturally occurring compound toxic to the insulin-producing beta cells in the pancreas and is commonly used to induce experimental diabetes in animal models, particularly rodents [8,9]. When streptozotocin is administered to animals, it causes hyperglycemia with elevated blood sugar level due to the loss of insulin secretion [10].

Using hyperspectral imaging (HSI), the wound bed and surrounding tissue can be analyzed to evaluate factors such as tissue oxygenation, granulation tissue formation, epithelialization, and collagen content by capturing the reflectance spectra of different tissue components such as hemoglobin, water, collagen, and lipids [11]. HSI further aided in identifying and quantifying inflammatory processes within wounds. Inflammatory markers, such as increased blood flow, edema, and specific biochemical signatures associated with inflammation, were detected and monitored using hyperspectral data [12]. Generally, HSI was applied to evaluate the efficacy of different wound treatments and interventions. As such, capturing spectral data before and after gas plasma treatment in different mouse models makes it possible to objectively assess changes in wound parameters, such as tissue oxygenation, inflammation levels, and overall wound healing progression [13,14,15].

Numerous treatment strategies target defective wound healing [16]. To optimize wound healing processes, medical gas plasma increasingly appears as a successful therapeutic approach [17]. Gas plasma is a partially ionized gas and a potent source of several gaseous reactive oxygen and nitrogen species (summarized as ROS), which are transported directly to the region of interest. ROS can stimulate cellular proliferation and migration, immune cell recruitment, and angiogenesis [18]. Based on these data, it was shown that gas plasma therapy not only heals rodent wounds but also patients benefit from gas plasma therapy safely and with antimicrobial efficacy [19]. Consequently, several clinical trials have indicated a promising role of medical gas plasma in treating extensive or non-healing diabetic wounds [20,21,22]. It is known that gas plasma-derived ROS act on skin cells [23] and participate in processes that are redox-regulated [24]. However, there is limited molecular understanding of how the gas plasma-derived reactive species mixtures may trigger adequate angiogenesis to stimulate healing. Therefore, we utilized a murine full-thickness ear incision model in an STZ-induced mouse model to investigate the effects of gas plasma therapy in diabetic wound healing.

## 2. Material and Methods

### 2.1. Animals and Wounding

**Figure 1:**
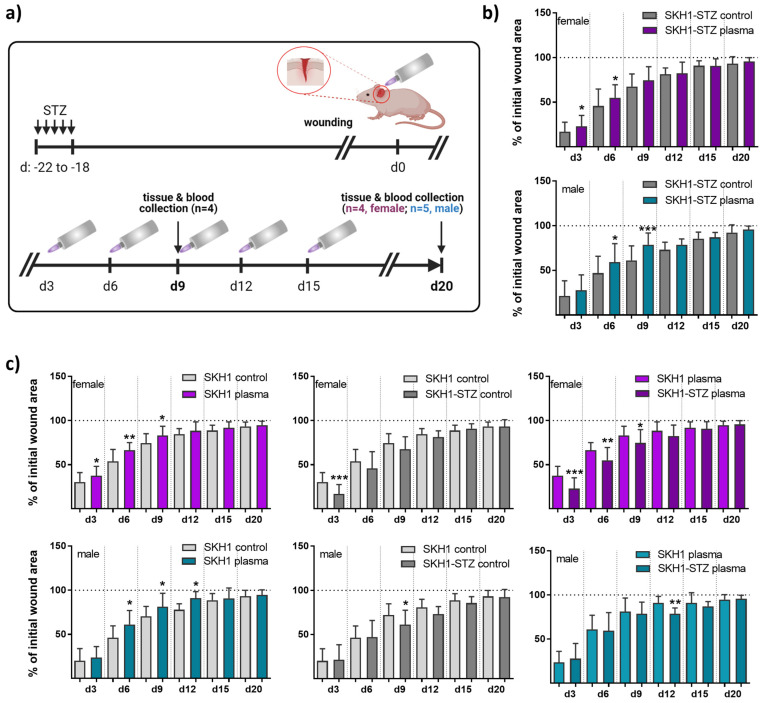
**Repeated gas plasma treatment shortened wound closure in diabetic mice.** (**a**) Schematic timeline of gas plasma treatment in diabetic mice illustrates wound closure and treatment regimes. STZ was i.p. administered on five consecutive days, and ear wounds were generated after 18 days (corresponding to d0). Afterward, wounds were gas plasma-treated (10 s) every third day (abbreviated as d). Wound tissue and blood were collected on d9 or d20, respectively. (**b**,**c**) The wound closure rate is plotted as the percentage reduction of the original wound area over time for gas plasma-treated females (**upper** panels, pink) and males (**lower** panels, blue) compared to the untreated controls in diabetic mice (**b**), in the SKH1 wild-type wound model (**c**, **left**), or when comparing untreated (**c**, **middle**) or gas plasma-treated (**c**, **right**) SKH1-STZ with SKH1 mice. (**c**) Data are presented as mean + SD; * *p* < 0.05, ** *p* < 0.01, and *** *p* < 0.001 compared to corresponding controls as indicated.

SKH1-hr hairless immunocompetent mice (Charles River Laboratories, Sulzfeld, Germany) aged 8–10 weeks, ~20 g each, were used for the wound healing studies under protocols by the local ethics committee according to the guidelines for care and use of laboratory animals (7221.3-1-044/16) and to the NIH Guide for the Care and Use of Laboratory Animals. They were housed with a 12-h light-dark cycle and free access to food and water. The overall study design describes the treatment regime and time points (Figure 1a). Immediately prior to injection, streptozotocin (STZ) was dissolved in 50 mM sodium citrate buffer to a final concentration of 4 mg/mL. STZ was administered intraperitoneal (i.p., 50 mg/kg) on five consecutive days. Under ketamine sedation (1.9 mg/mouse) and xylazine (0.19 mg/mouse, i.p.) on the experimental day of wounding (d0, after appr. 20 days), blood glucose levels were monitored (criterion for successful diabetes induction was >8.3 nmol/L). Additionally, we used the non-diabetic background strain SKH as a control for plasma treatment (n = 4 males, n = 5 females for d9, and n = 3, n = 4 females for d20). Afterward, under anesthesia, wounding on both ears using a microscissor was performed as previously described by removing the upper epidermal and dermal layers [25]. The area of the full-thickness dermal wound was in the range of 2.5–4.0 mm^2^. The wound tissue was recovered at specific time points of sacrifice, i.e., 9 and 20 days after injury, to analyze a total of 40 mice.

### 2.2. Medical Gas Plasma Treatment and Wound Closure Measurements

An atmospheric pressure argon plasma jet (*kINPen MED*; neoplas, Greifswald, Germany) was used, which ionized a flow (5 standard liters per minute) of argon gas (purity 99.9999; Air Liquide, Bremen, Germany) at 1 MHz. The device has been approved as a medical product (today: class IIb) in Germany and Europe since 2013 [19]. Gas plasma treatment of wounds of both ears was performed under anesthesia using the tip of the gas plasma effluent (i.e., conductive mode [26]) at a constant distance of 8 mm using an autoclavable spacer. Only the wound region and not the surrounding ear was exposed to the plume of the gas plasma jet. Ear wounds were gas plasma-treated for 10 s every third day or, in another group of animals, were left untreated (ctrl) over 9 (four times gas plasma-treated or left untreated) or 20 (six times gas plasma-treated or left untreated) days. Experiments were terminated and animals were sacrificed either on day 9 or day 20. Wound closure was measured on the day of wounding (d0) and every third day after that. Ex vivo, skin cells were indirectly gas plasma-treated, as previously described [23].

### 2.3. Hyperspectral Imaging (HSI) System

To analyze the tissue microcirculatory characteristics in real-time, the HSI camera system *TIVITA Tissue* (Diaspective Vision, Am Salzhaff, Germany) was utilized as previously described [13]. Then, the light of the visible and invisible spectrum (500–1000 nm) is analyzed, attributing sub-spectra to distinct parameters, such as oxygen saturation (StO_2_) in superficial layers and the perfusion into deeper skin regions (near-infrared index, NIR; 4–6 mm). In addition, the software can analyze the hemoglobin distribution using a tissue hemoglobin index (THI) and the water content using a tissue water index (TWI). Hyperspectral images were acquired under standardized conditions with a distance from the camera to the ear of 50 cm. After wounding, HSI parameters were immediately (within 10 min) recorded after gas plasma treatment and compared to that of untreated control wounds (set to 1). This was repeated every third day up to the endpoint (d20) in control or gas plasma-treated wounds (again, within 10 min post-exposure). For data analysis, the camera-specific software *TIVITA Suite* 1.0 (Diaspective Vision, Am Salzhaff, Germany) was used to calculate all parameters in well-defined circular wound regions (n > 4).

### 2.4. Skin Cell Isolation and Homogenization of Ear Tissue

Primary skin cells were isolated from diabetic in vivo gas plasma-treated or untreated skin tissue by enzyme-mediated removal and digestion of the epidermal and dermal layers according to the instructions of an epidermis dissociation kit (Miltenyi Biotec, Teterow, Germany). Afterward, using an octaMACS dissociator, the cell suspension was homogenized in gentleMACS C tubes to obtain live cells after being passed through a 7 µm MACS SmartStrainer (Miltenyi Biotec, Teterow, Germany). Skin cells were cultured over ten days in EMEM medium (PromoCell, Heidelberg, Germany) supplemented with 10% fetal bovine serum, 1% penicillin/streptomycin, and L-glutamine (Sigma-Aldrich, Taufkirchen, Germany) in a humidified incubator at 37 °C with 5% CO_2_. Ear tissue from right ears was collected on days 9 and 20. Briefly, fresh tissues from ears were removed, snap-frozen in liquid nitrogen, and stored at −80 °C. Homogenization was performed in RNA lysis buffer (Bio&Sell, Feucht, Germany) for transcriptional expression analysis or in RIPA buffer containing protease and phosphatase inhibitors (cOmplete Mini, phosSTOP, PMSF; Sigma-Aldrich, Taufkirchen, Germany) to analyze protein levels and activity using a FastPrep-24 5G homogenizer (MP biomedicals, Eschwege, Germany).

### 2.5. RNA Extraction and Quantitative PCR Analysis

To quantify mRNAs by quantitative PCR (qPCR), 1 μg of RNA was transcribed into cDNA, and qPCR was conducted in duplicate using SYBR Green I Master (Roche Diagnostics, Mannheim, Germany). Gene-specific primers were used (BioTez, Berlin, Germany) (Table A1). The housekeeping genes *GAPDH* and *RPL13A*, whose expression was unaffected by gas plasma treatment, were used as an internal control for normalization. Gene expression was analyzed using the 2^−∆∆Ct^ method.

### 2.6. Protein Analyses Using WES Quantification System or Immunohistochemical Staining

**Figure A1:**
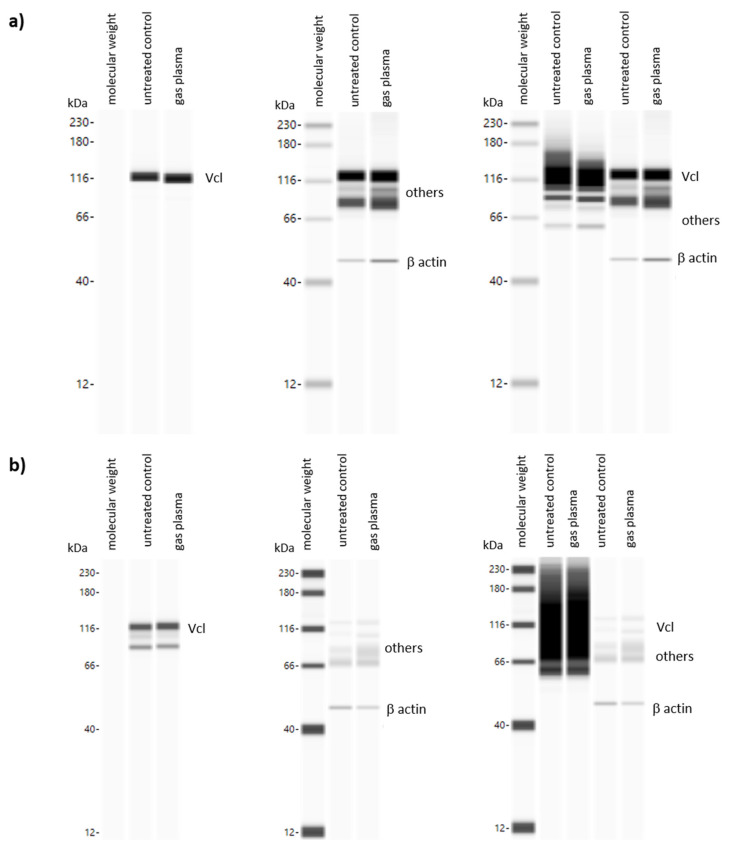
Western blot analysis using WES system of (**a**) vinculin (Vcl, **left** image), b actin (**middle** image, housekeeping protein) and both proteins together (**right**) in gas plasma-treated vs. untreated mice on d9; (**b**) vinculin (Vcl, **left** image), b actin (**middle** image, housekeeping protein) and both proteins together (**right**) in gas plasma-treated vs. untreated mice on d20. Band intensities were quantified using Compass 8.0 Software (ProteinSimple, Wiesbaden, Germany).

Protein targets were quantified based on their significance within the main cellular responses to angiogenesis. These included molecules of the adherence junctions (e.g., Fak, pFak, and vinculin) and angiogenesis-related targets (e.g., iNOS, syndecan, and cytokeratin). Western blot analysis was performed using WES according to the manufacturer’s instructions. Band intensities (Figure A1) were quantified using Compass Software 6.0 (ProteinSimple, Wiesbaden, Germany) and expressed as fold change compared to the corresponding control. GAPDH served as a housekeeping protein (all antibodies were from Cell Signaling Technologies, Heidelberg, Germany). On days 9 and 20, wound regions of the left ears were collected and fixed in 4% paraformaldehyde (Sigma-Aldrich, Taufkirchen, Germany) overnight. Paraffin blocks were cut into 5 µm-sections using a microtome to retrieve tissue sections that were stained with hematoxylin and eosin (H&E; Carl-Roth, Karlsruhe, Germany). Tissues were immunohistochemically stained with Fak, pFak, and Sdc primary antibodies using a SignalStain boost IHC detection reagent (all Cell Signaling Technologies, Heidelberg, Germany). Proteins in epidermal and dermal layers were quantified using ImageJ 1.54 software. In addition, tissue samples were incubated with fluorescently labeled (Alexa Fluor 488 or Alexa Fluor 647) primary antibodies targeting iNOS and cytokeratin (all Cell Signaling Technologies, Heidelberg, Germany). Additionally, skin cells were fixed in 4% paraformaldehyde (Sigma-Aldrich, Taufkirchen, Germany) for 20 min, washed, permeabilized with Triton X-100 (0.01% in PBS; Sigma-Aldrich, Taufkirchen, Germany), and stained with antibodies targeting vinculin. All tissue and cell slides prepared for immunofluorescence analysis were stained with DAPI (4′,6-diamidino-2-phenylindole) to counterstain nuclei, followed by mounting samples onto glass microscope slides using a mounting medium (VectaShield; Biozol, Eching, Germany) prior to analysis using an Axio Observer Z.1 microscope (Zeiss, Jena, Germany).

### 2.7. Bead-Based Cytokine and Chemokine Profiling in Blood Serum and Protein Lysates

Blood was collected retrobulbarly in EDTA tubes at days 0, 9, and 20 and centrifuged, and the serum supernatant was stored at −80 °C until use. The inflammatory secretion profiles in the blood serum of diabetic SKH1 mice were measured using multiplex cytokine detection technology (LegendPLEX; BioLegend, Amsterdam, The Netherlands) according to the manufacturer’s instructions. Briefly, the bead-based sandwich immunoassay was measured using flow cytometry (CytoFLEX LX; Beckman-Coulter, Krefeld, Germany) analyzing fluorescently labeled antibodies targeting granulocyte-macrophage colony-stimulating factor (GM-CSF), tumor necrosis factor (TNF) α, interferons (IFN) α and β as well as γ, four chemokines (monocyte chemotactic protein (MCP1 or CCL2), chemokine ligand 5 (RANTES or CCL5), C-X-C motif chemokine 10 (IP10 or CXCL10), and 1 (KC or CXCL1), and four interleukins (IL1β, IL6, IL10, and IL12p70). Appropriate data analysis software (LegendPLEX 8.0 software; BioLegend, Amsterdam, The Netherlands) was utilized for target quantification.

### 2.8. Statistical Analysis

All experiments were done with tissue of at least three animals per experimental group. In vitro assays were repeated three times independently. Data show mean + standard deviation if not indicated otherwise, and data were statistically compared with *p* values indicated by * *p* < 0.05, ** *p* < 0.01, or *** *p* < 0.001. Graphing and statistical analysis were performed with *prism* 7.04 (GraphPad Software, San Diego, CA, USA) using analyses of variances (ANOVA).

## 3. Results

### 3.1. Gas Plasma-Treated Diabetic Wounds Showed Faster Healing Responses

The diabetogenic substance streptozotocin (STZ) was multiply injected at low doses at five consecutive days into immunocompetent nude SKH1 mice to provoke a diabetic type 1 mouse model. The mice developed characteristics of human type 1 diabetes mellitus (DM1) due to the selective destruction of pancreatic islet β-cells [9]. Here, the model was used to study medical gas plasma’s potential to promote healing in a dermal full-thickness ear wound model (Figure 1a). Diabetic mice were either treated with gas plasma (10 s) every third day or were left untreated (ctrl) over 9 (d9) or 20 (d20) days. On the day after injury, the size of the remaining defect areas (as percentage of the original wound size) was examined, demonstrating a similar morphology and size of all wounds. Single quantitative analysis at each healing interval showed significantly accelerated wound closure on days 3 and 6 in females and 6 and 9 in males compared to controls. This demonstrated a quicker wound size reduction and suggested improved re-epithelialization in the gas plasma-treated compared to the untreated wounds. However, the original wound was covered completely with epithelium approximately on day 9. At two weeks after the injury, no difference was found, and near-complete wound closure (>95%) was achieved on day 20 in all experimental groups (Figure 1b). Additionally, we compared healing responses regarding wound closure time with each other in both gender with all possible treatment and control groups including the non-diabetic SKH1 background strain. Gas plasma treatment shortened the time of wound closure (left diagrams) in females (upper panel, pink) and males (lower panel, cyan). Improved wound closure was found in non-diabetic compared to diabetic mice. Additionally, wound closure rates were lower in diabetic compared to non-diabetic mice receiving gas plasma therapy, validating the model (Figure 1c).

### 3.2. Gas Plasma-Treated Wounds Showed Different Microcirculatory Parameters

**Figure 2:**
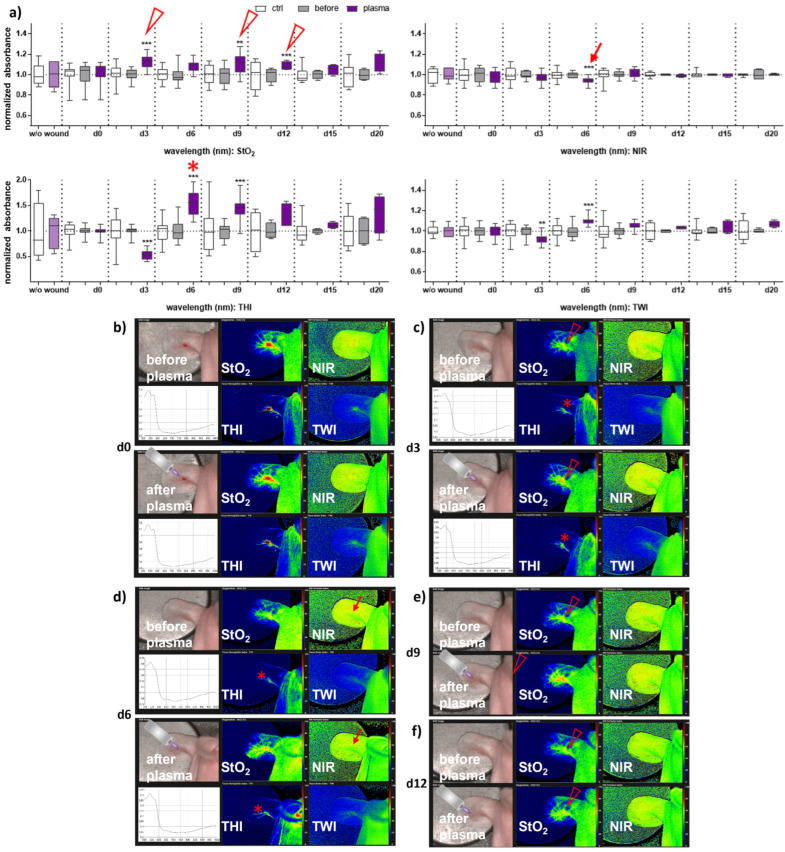
**Gas plasma treatment altered microcirculatory parameters during wound healing in females.** (**a**) Quantification of hyperspectral imaging parameters of ear wounds exposed to gas plasma, including tissue oxygenation (StO_2_), tissue hemoglobin index (THI), perfusion in deeper layers (NIR), as well as the tissue water index (TWI). Apparent changes of StO_2_ (arrowhead), THI (star), and NIR (arrow) are indicated. (**b**–**e**) Representative false-colored images of murine ear wounds exposed to gas plasma for 10 s before (**upper** panels) and after treatment (**lower** panels) in females on d0 (**b**), d3 (**c**), d6 (**d**), d9 (**e**), and d12 (**f**). Results are expressed as boxplots ± SEM of at least six independent measurements, and statistical analysis was performed using one-way analysis of variances with ** *p* < 0.01 and *** *p* < 0.001.

**Figure 3:**
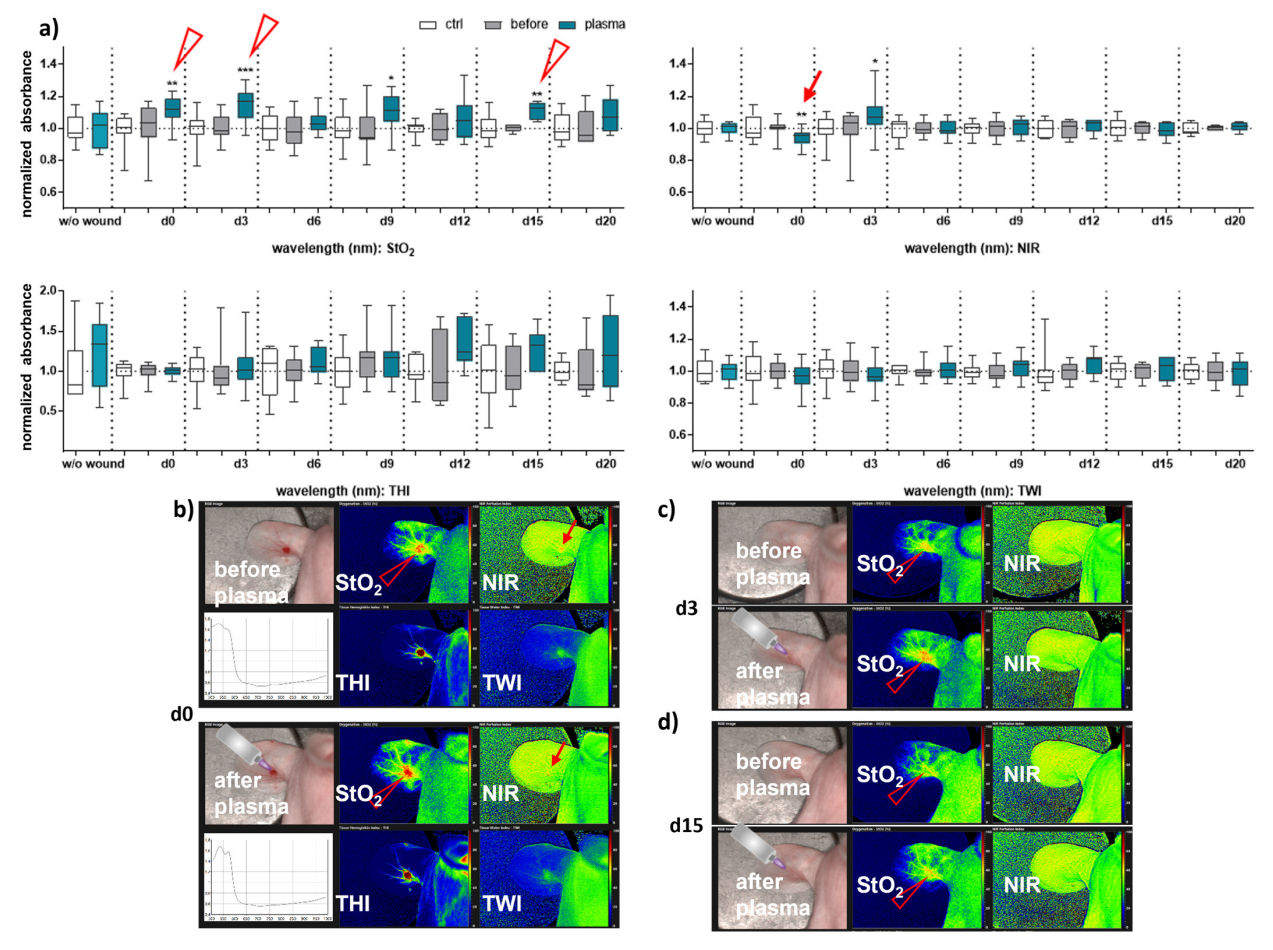
**Gas plasma treatment modulated microcirculatory parameters during wound healing in males.** (**a**) Quantification of hyperspectral imaging parameters of ear wounds exposed to gas plasma, including tissue oxygenation (StO_2_), the tissue hemoglobin index (THI), perfusion in deeper layers (NIR), as well as the tissue water index (TWI). Apparent changes of StO_2_ (arrowhead) and NIR (arrow) are indicated. (**b**–**d**) Representative false-colored images of murine ear wounds exposed to gas plasma for 10 s before (**upper** panels) and after treatment (**lower** panels) in males on d0 (**b**), d3 (**c**), and d15 (**d**). Results are expressed as boxplots ± SEM of at least six independent measurements, and statistical analysis was performed using one-way analysis of variances with * *p* < 0.05, ** *p* < 0.01, and *** *p* < 0.001.

Next, we aimed to evaluate HSI for monitoring gas plasma-induced microcirculatory wound parameter changes in diabetic mice. HSI showed oxygen saturation levels throughout all sampling time points, with slight differences between females and males. Superficial StO_2_ (tissue oxygenation levels) were significantly enhanced with gas plasma therapy on days 3, 9, and 12 in females (Figure 2a), whereas StO_2_ improved already on the wounding day (d0) and days 3, 9, and 15 in males (Figure 3a). The NIR index was mostly unchanged from untreated controls and before plasma treatment. However, it showed slightly decreased perfusion on d6 in females and on the day of wounding (d0) in males after plasma treatment. This decreased perfusion was significantly increased three days later (d3) in males, suggesting gender-specific differences in oxygenation and deeper perfusion. For the tissue hemoglobin index THI and tissue water index TWI, gas plasma treatment caused an increase in males to a significantly smaller extent than in females (Figure 2 and Figure 3a). HSI parameters were calculated as false-colored images. Representative measurements are shown for time points where HSI parameters significantly differed from untreated controls. HSI parameters were first shown on the wounding day after single gas plasma treatment in females (Figure 2b) and males (Figure 3b) compared to the situation before plasma treatment. Besides the alterations mentioned above of oxygenation in females, THI was strongly decreased on d3 (Figure 2c). In contrast, we found a strong THI increase on d6 and d9 and decreased NIR index on d6 (arrow, Figure 2d). Significantly enhanced StO_2_was further shown during later time points after wounding in females (Figure 2e,f) and males on d3 (Figure 3c) and d15 (Figure 3d). HSI parameter measurements suggested strong oxygenation and surface perfusion differences that were dependent on wound healing stage and, to a lesser extent, on gender.

### 3.3. Gene and Protein Expression of Angiogenic Factors in Newly Regenerated Tissue of Healing Wounds Were Significantly Altered with Gas Plasma Therapy

**Figure 4:**
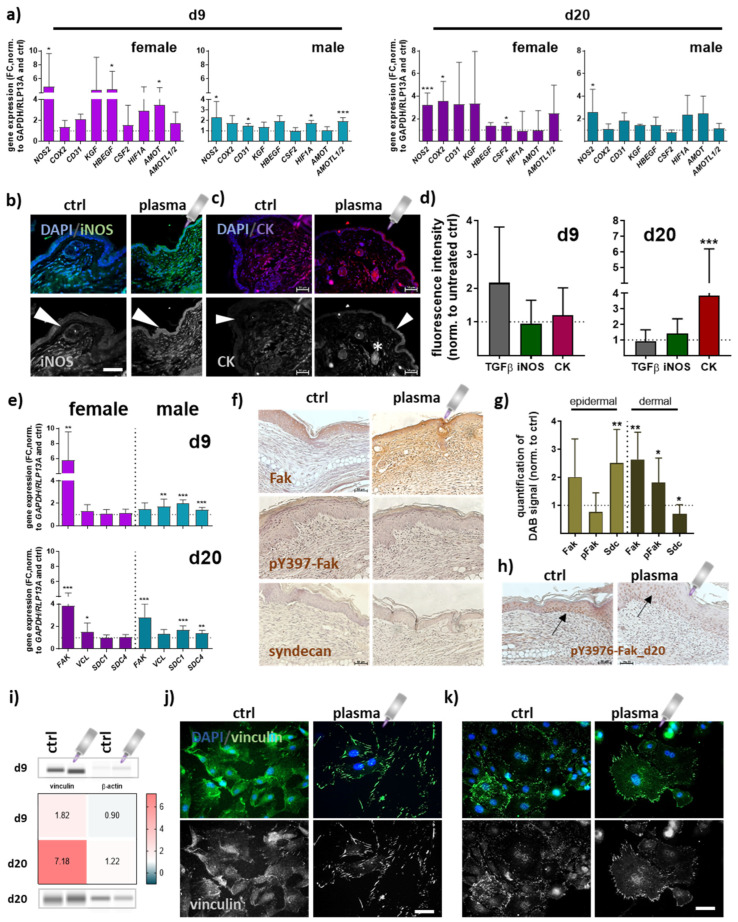
**Gas plasma exposure promoted the expression of angiogenic targets controlling neovascularization and angiogenesis.** (**a**) Mice were treated with gas plasma as described. Gene expression analysis of angiogenetic targets (e.g., *NOS2*, *COX2*, *CD31*, *KGF*, *HBEGF*, *CSF2*, *HIF1A*, *AMOT*, and *AMOTL1/2)* was performed with total RNAs isolated from the wound regions on endpoints (d9/d20). (**b**,**c**) Immunofluorescence analysis of iNOS (**b**) and cytokeratin (CK) (**c**) on d20 after injury. (**d**) Quantification of fluorescence signal intensities at d9 and d20. (**e**) Gene expression analysis of targets of focal adhesions (e.g., *FAK*, *VCL*, and *SDC1/4*). (**f**) Immunohistochemical DAB-staining of Fak, pY397-Fak, and syndecan in controls (**left**) and following gas plasma treatment on d9. (**g**) Quantification of DAB signals in the epidermal and dermal layer of re-epithelialized areas on d9. (**h**) DAB staining of Y397-Fak in untreated and gas plasma-treated mice on d20. (**i**) Protein expression levels of vinculin and β-actin were quantified using WES analysis on d9 (**upper** panel) and d20 (**lower** panel) compared to untreated mice. The western blot bands are cropped from Figure A1, where also molecular weight labels can be found. (**j**,**k**) Immunofluorescence analysis of vinculin in skin cells isolated from untreated (**j**) and gas plasma-treated SKH1-STZ (**k**) mice. Cells were isolated, cultivated, and stained for vinculin in gas plasma-treated (**right** images) and compared to untreated (**left** images) skin cells in vitro. Males and females were used on d9 and d20. Data are presented as mean + SD with * *p* < 0.05, ** *p* < 0.01, and *** *p* < 0.001, as compared to controls (ctrl). Scale bar is 50 µm, the grey symbol represents the plasma jet.

Quantitative PCR experiments were conducted to elucidate gene expression profiles of repair-related genes in angiogenesis at early (d9) and late stages (d20) of wound healing. Overall, data indicated an apparent difference between gas plasma- and untreated mice. Significantly increased transcriptional levels were found for inducible nitric oxidase (*NOS2 or iNOS*) and cyclooxygenase (*COX2*). Growth factors (*KGF*, *HBEGF*, *CSF2*) were mainly upregulated in females in contrast to males. Hypoxia-induced factor 1 alpha (HIF1A) mRNA expression also significantly increased in gas plasma-promoted healing tissue at day 9. The angiomotin family members *AMOT and AMOTL1/2* mRNA expression was significantly higher at days 9 and 20 in females and males in gas plasma groups (Figure 4a). The protein expression of iNOS in epidermal and dermal layers was reduced in non-treated compared to gas plasma-treated mice (Figure 4b). Cytokeratins (CK) play a crucial role in maintaining cell structure, integrity, and mechanical stability, and have been detected in keratinized epidermis [27], and certain non-epithelial cells, including endothelial cells involved in angiogenesis [28]. Distribution of cytokeratin (red) was shown by immunofluorescence staining, demonstrating a significantly increased expression in epidermal layers (arrow) and in the endothelial cells surrounding blood vessels (stars) following gas plasma treatment (Figure 4c) on d20 (Figure 4d). During healing, focal adhesion kinase (Fak) and vinculin become activated and play a key role in regulating cellular processes necessary for wound closure [29]. *FAK* expression was significantly higher on both healing intervals obtained, whereas vinculin (*VCL*) mRNA levels were overall similar in both genders. Syndecans (Sdc) are cell-surface proteoglycans expressed in differentiating keratinocytes and transiently upregulated in all epidermis layers upon tissue injury [30]. *SDC1/4* levels were significantly upregulated in males but unchanged on both time points in females in gas plasma groups (Figure 4e). To address the targets’ cellular distribution and expression, Fak, phosphorylated pY397-Fak, and syndecan expressing epidermal or dermal cells were further investigated using immunohistochemical staining. First, we validated the strong gas plasma-induced effect on Fak expression, whereas pY397-Fak was mainly increased in dermal cells. Syndecan expression was upregulated in epidermal cells but down-regulated in the dermal tissue layer on d9 (Figure 4f,g), as shown in representative images (Figure 4f). On the endpoints (d20), we found no expression level differences with gas plasma treatment. However, pY397-Fak expression was higher overall in the epidermis in both groups (arrows, Figure 4h). Vinculin, a structural adaptor protein of focal adhesions, was quantified and found upregulated with gas plasma treatment (Figure 4i). Interestingly, we obtained a strong enrichment of vinculin in leading-edge lamellipodia and filipodia. Non-treated skin cells (left images in j) displayed diffuse vinculin staining with some vinculin-positive staining of focal adhesions, whereas in vitro gas plasma-treated skin cells showed strong vinculin-positive cellular protrusions staining (images in Figure 4j). In vivo gas plasma-exposed skin cells affirmed this switch in focal adhesion dynamics (Figure 4k) suggesting a gas plasma-supporting effect on the cellular architecture as also previously described in dermal fibroblasts [31].

**Figure 5:**
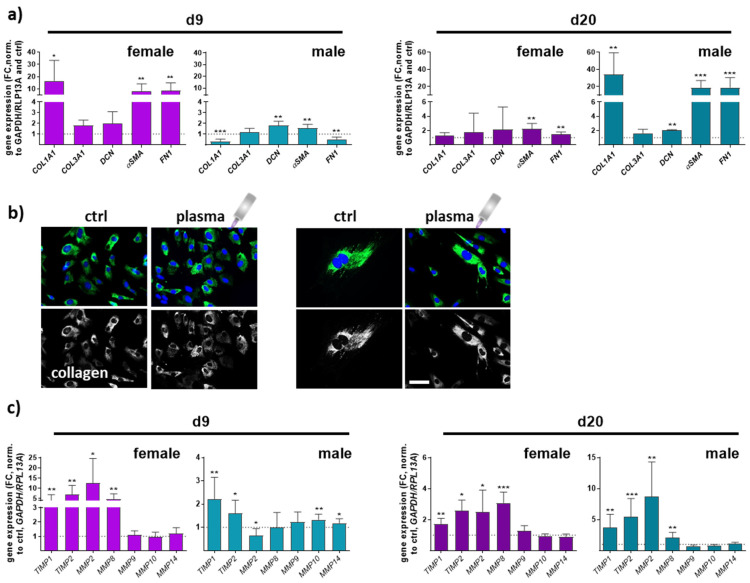
**Gas plasma exposure modulated the expression of ECM components and MMP targets controlling ECM degradation.** (**a**) Mice were treated with gas plasma as described. Gene expression analysis of ECM components (e.g., *COL1A1*, *COL3A1*, *DCN*, *αSMA*, and *FN1*) was performed with total RNA isolated from wound regions on endpoints (d9/d20). (**b**) Immunofluorescence analysis of collagen in skin cells isolated from untreated and gas plasma-treated SKH1-STZ mice. Cells were isolated, cultivated, and stained for vinculin in gas plasma-treated (**right** images) and compared to untreated (**left** images) skin cells in vitro. (**c**) qPCR of MMPs (*MMP2/8/9/10/14*) and their inhibitors (*TIMP1/2*). Males and females were used on d9 and d20. Data are presented as mean + SD with * *p* < 0.05, ** *p* < 0.01, and *** *p* < 0.001, as compared to controls (ctrl). Scale bar is 50 µm, the grey symbol represents the plasma jet.

Collagen type I alpha 1 (*COL1A1*), collagen type III alpha 1 (*COL3A1*), and decorin (*DCN*), a small leucine-rich proteoglycan, are major extracellular matrix (ECM) components of dermal skin tissue. Data from females and males displayed a significant effect on collagen 1 expression compared to collagen 3 levels. *COL1A1* expression in females was higher on day 9 but decreased on d20 in contrast to males, where the effect was the opposite. No significant differences were found between all groups regarding *COL3A1* gene expression. Decorin functions in collagen fibrillogenesis and was significantly upregulated after gas plasma exposure in males compared to a slight increase in females. Next, we quantified the expression of smooth muscle actin α (*αSMA*) and fibronectin 1 (*FN1*), which were strongly upregulated with gas plasma treatment on both time points, except for FN1 in males on d9. Interestingly, females (left diagrams) responded earlier with an upregulation of ECM targets on d9 compared to males (right diagrams) in which these targets were strongly upregulated on d20, affirming gender-specific differences in the expression of ECM-associated targets (Figure 5a). To show the gas plasma effects on collagens in vitro, skin cells of untreated and gas plasma-treated SKH1-STZ mice were isolated, cultivated, and fluorescently stained for collagen I, validating the in vivo results. In vivo pre-treated cells showed stronger staining than non-treated skin cells, which was once again stronger than in untreated controls (Figure 5b). ECM remodeling is essential in forming new blood vessels from pre-existing ones. Fibroblasts produce matrix metalloproteinases (MMPs), a group of enzymes involved in the breakdown of ECM components during wound healing. MMPs (MMP2, 8 (collagenase 2), 9 (gelatinase B), 10 (stromelysin 2), 14) were differentially regulated, including partial differences between genders. MMP2 and MMP8 were mainly upregulated except for males on d9, but their overall relative expression was up to 5 times higher on d9 in gas plasma-treated animals. MMP8 is primarily associated with acute inflammatory processes, underlining its lower expression on d20. MMPs 9, 10, and 14 were partly upregulated in males on d9, suggesting transient degradation regulation and remodeling of the ECM components following gas plasma treatment. Additionally, tissue inhibitors of MMPs, *TIMP1* and *2*, were upregulated at all time points in both genders (Figure 5c,d).

### 3.4. Medical Gas Plasma Modified Systemic Cytokine and Growth Factor Levels

**Figure 6:**
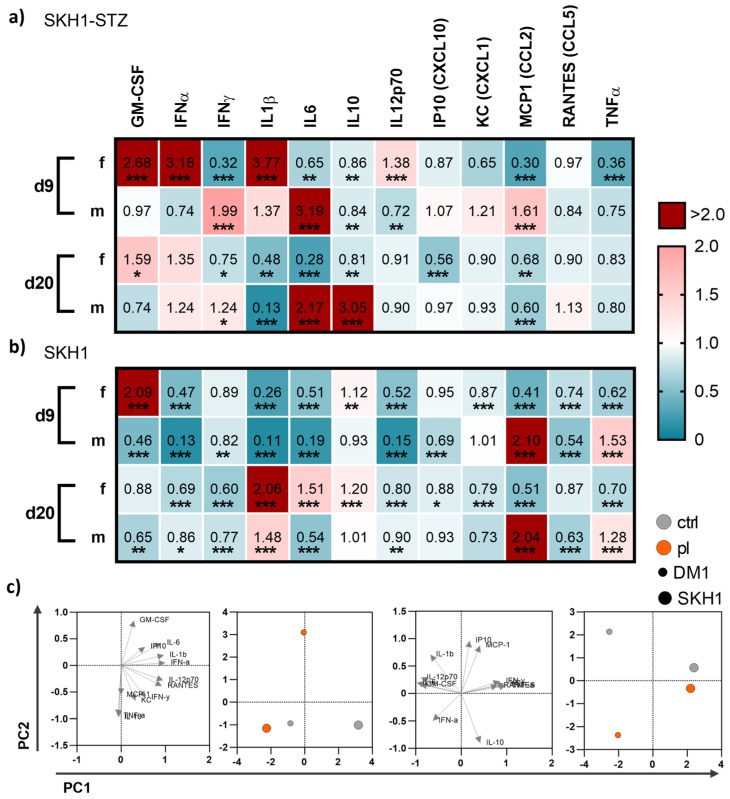
**Gas plasma exposure altered in vivo cytokine patterns.** (**a**,**b**) Diabetic type 1 (SKH1-STZ; **a**) and background (SKH1; **b**) mouse models were treated with gas plasma and compared to their corresponding untreated controls. Heat maps show medians and indicate growth factor (GM-CSF, TNFα), cytokine (IFNα/β/γ, IL1β/6/10/12p70), and chemokine (IP10, KC, MCP1, RANTES) levels in the blood normalized to corresponding untreated control (ctrl) wounds. (**c**) Principal component analysis (PCA) calculated from cytokine concentrations sowing loads (left) and PC scores (right). Data are based on untreated (grey) and gas plasma-treated (orange) sacrificed on d9 (left) and d20 (right) in SKH1-STZ (small circle) versus SKH1 model (big circle, n ≥ 4). Statistical analyses were conducted using one-way analysis of variance with * *p* < 0.05, ** *p* < 0.01, and *** *p* < 0.001; d = day; f = female; m = male.

To understand whether local gas plasma therapy has a system impact on inflammatory mediators, we mapped cytokine and growth factor levels in blood plasma on days 9 and 20 post-wounding in diabetic and non-diabetic mice. We found differences between both endpoints of measurements as well as the mouse model, respectively (Figure 6). On d9, gas plasma exposure had a more pronounced effect on the secretion profile in diabetic females than in diabetic males. In particular, GM-CSF, IFNα, IL1β, and IL12p70 were significantly upregulated in females but down-regulated in males, whereas IFNγ, IL6, and MCP1 were increased in males in contrast to females. Changes in the cytokine and chemokine release were found for IL6 and IL10 (significantly increased, red) and IL1β (significantly decreased, blue) in males on d20 but not in females (Figure 6a), suggesting that the repeated, gas plasma-induced inflammatory response is differentially regulated on a systemic level in females. In non-diabetic wounded SKH1 mice, the differences between females and males were less prominent for the secretion levels of GM-CSF, MCP1, and TNFα on d9, and IL6 and MCP1 on d20, respectively. The data indicated a modest but visible systemic effect of gas plasma treatment with differences between females and males (e.g., GM-CSF, IL1β, MCP1, and TNFα) along with similar tendencies on d9 and d20 in both mouse models (Figure 6b). Principal component analysis (PCA) calculated from absolute mediator concentrations highlighted differences in blood serum levels of some of these cytokines or chemokines. However, their overall regulation was mostly consistent between gas plasma-treated and untreated mice, as underlined by the PCA analysis (Figure 6c).

## 4. Discussion

**Figure 7:**
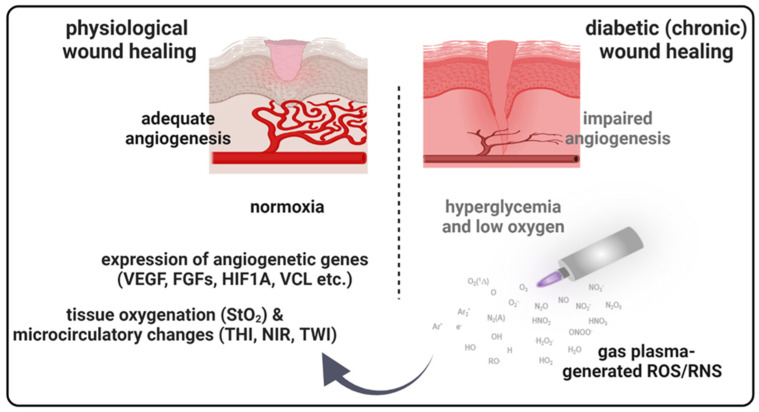
**Schematic overview of gas plasma-induced effects on the microcirculatory system and angiogenic gene expression in diabetic wound healing.** Due to a postponed, incomplete, or uncoordinated healing process, diabetic wounds are characterized by a persistent inflammatory phase associated with an impediment in the formation of mature granulation tissue and vascular damage resulting in low oxygen content and obstruction of wound closure. Gas plasma-generated reactive oxygen species (ROS) support diabetic wound closure due to the promotion of pro-angiogenic gene transcription and stimulating microvascular parameters like the oxygenation of superficial skin tissue (StO2) to support angiogenesis in diabetic wound models. THI, tissue hemoglobin index; NIR, near-infrared index; TWI, tissue water index.

Diabetes can negatively impact multiple wound healing stages, leading to chronic wounds and an increased complication risk [32]. New therapeutic approaches are needed to reduce diabetic wounds’ occurrence rates by 50% in patients [32]. The idea that reactive oxygen species (ROS) or ROS-modulating technologies, including gas plasma [17], improve wound healing was proposed before [33]. ROS are key modulators in the orchestration of physiological wound healing. They regulate immune cell recruitment [34,35], angiogenesis [36,37], and optimal blood perfusion [38]. Although there are many studies on novel techniques for treating diabetic wound healing, efficacies are often modest, and molecular mechanisms mostly remain unclear. Using a murine diabetic wound model, we here investigated medical gas plasma and determined molecular targets of improved healing and advanced wound healing parameter monitoring (Figure 7).

Streptozotocin (STZ) is selectively taken up by β-cells via glucose transporters and causes DNA damage, leading to β-cell death and subsequent impairment of insulin production [9]. This property makes STZ a useful tool for creating animal models that mimic certain aspects of human diabetes, particularly type 1 diabetes mellitus (DM1). STZ has been widely used in preclinical research to investigate the pathogenesis of diabetes to test novel therapeutic approaches and evaluate the efficacy of potential treatments [39,40,41]. However, it is essential to carefully consider the limitations and differences between animal models and human diabetes when interpreting the results obtained from STZ-induced studies. In our preclinical study, we confirmed the poorer healing of diabetic wounds in untreated as well as gas plasma-treated STZ-induced diabetic compared to non-diabetic mice (Figure 1). Nevertheless, gas plasma treatment shortened the diabetic wound closure and suggested improved re-epithelialization in early wound phases (Figure 4). Our data confirmed slightly better healing rates in females, as described in previous studies in mice [26,42]. We could not fully explore the gender dimension of the molecular mechanisms on wound healing to possibly explain different healing responses with gas plasma therapy that was seen in female versus male mice. The gender differences in wound healing rates may be related to mutual cross-talk between redox and hormone signaling [43], as demonstrated before [42,44,45]. The modulatory effects of hormone levels affected the immune response [46] together with a modulated cytokine expression, resulting in pro- and anti-inflammatory characteristics [47]. Intriguingly, our data showed that differential healing rates in both genders are associated with a specific cytokine expression and secretion pattern. In particular, IFNα, IL1β, and IL12p70 were upregulated in animals receiving gas plasma therapy, suggesting a transiently wound healing-related effect of gas plasma on early time points in females. Compared to this, IFNγ, a critical activator of macrophages, IL6, and CCL2 (MCP1) were increased in males at the first endpoint of the measurement but not in females. Additionally, we observed increased systemic IL6 and IL10 levels up to d20 after injury in males, which provides a potential explanation for the gender-specific healing rates. In summary, blood cytokines may predict healing rates, which was also suggested in another study [48].

There is also evidence that gas plasma directly promotes angiogenesis, a process by which new blood vessels are formed from pre-existing ones [31,49,50,51]. To validate the gas plasma-supported vascular network formation finding in our diabetic wound model, we applied hyperspectral imaging (HSI) to provide valuable insights into the biochemical composition and physiological changes occurring in the wound site over time. The validity of this approach is based on previously published evidence in humans [14,15,52,53,54]. The HSI system has been developed for non-invasive integrative wound analysis with the device TIVITA Tissue System for qualitative and quantitative data interpretations [55,56]. It can be used in various applications [57,58]. In wound healing, HSI was used to evaluate the efficacy of different treatments and interventions [11]. By capturing spectral data before and after gas plasma treatment in different rodents [59] and diabetic models [60,61], it is possible to objectively assess changes in wound parameters, such as tissue oxygenation, inflammation level, and overall wound healing progression. This information can guide treatment decisions and optimize patient care. First, by analyzing the spectral characteristics of wounds, it is possible to estimate tissue oxygen saturation (StO_2_) and assess the oxygen supply to the wound bed. Changes in StO_2_ over time were shown in assessing wound healing progress and evaluating the effectiveness of gas plasma treatments in both genders, particularly in the first week after injury [13,31]. Our HSI parameter measurements suggested strong oxygenation and surface perfusion differences that were dependent on wound healing stage and, to a lesser extent, on gender. Secondly, HSI can analyze the wound bed and surrounding tissues to evaluate factors such as granulation tissue formation, re-epithelialization, and collagen content. By capturing the reflectance spectra of different tissue components, it is possible to determine the presence and distribution of specific biochemical constituents, such as hemoglobin, water, collagen, and lipids, which can provide information about wound healing progression [62]. The finding of a gas plasma-mediated increase of collagen fibers in re-epithelialized regions leading to an increased formation of granulation tissue underlines this aspect [31]. Moreover, HSI can aid in identifying and quantifying inflammatory processes within wounds. Inflammatory markers, such as increased blood flow, edema, and specific biochemical signatures associated with inflammation, can be detected and monitored using hyperspectral data [12]. As shown in bead-based cytokine and chemokine profiling, the repeated, gas plasma-induced inflammatory response is differentially regulated on a systemic level in both genders. By analyzing the spectral signatures of bacteria or microbial metabolites, HSI can identify the presence of infection and differentiate between infected and non-infected wounds [63]. Overall, hyperspectral imaging offers a non-invasive and objective approach to assess wound healing processes in diabetic wound healing in mouse models.

Gas plasma induces the upregulation of a multitude of growth factors and angiogenic transcripts in human keratinocytes, a finding confirmed by our in vivo-derived data (e.g., KGF, CD31, iNOS, HBEGF, CSF2, AMOT, and AMOTL1/2). In support of our findings, gas plasma treatment improved diabetic wound closure in conjunction with KGF receptor activation, a process central during wound healing [64]. The interactions of endothelial cell-cell adhesion molecules (PECAM-1 or CD31) are important in forming new vessels, as shown by augmented CD31 expression [65]. Gas plasma-released nitric oxide (NO) [66,67] may be a driver for the increased inducible NO synthase (iNOS) expression in gas plasma-treated wounds [68]. Lacking iNOS retards macrophage invasion and its expression of fibrogenic components that might further impair the fibrogenic behavior of fibroblasts [69]. The present study further suggests that the granulocyte-macrophage colony-stimulating factor (GM-CSF or CSF2) is required in cutaneous wound healing, particularly at early stages. GM-CSF is a cytokine that promotes angiogenesis during wound healing by stimulating endothelial cell proliferation [70] and migration [71,72], increasing vascular permeability [73], inducing the production of angiogenic factors [74], and recruiting pro-angiogenic cells [75]. These effects collectively contribute to forming a new vascular network, supporting tissue repair and regeneration. Moreover, angiomotin (AMOT and AMOT-like protein 1/2, AMOTL1/2) has been found to play multiple roles in angiogenesis and blood vessel formation, influencing endothelial cell behavior [76], VEGF signaling [77], endothelial junctions [78], and vascular permeability [79], in line with our findings. Cytokeratin was found to be significantly expressed in keratinocytes and around blood vessels in dermal gas plasma-treated skin layers. While cytokeratins are primarily associated with epithelial tissues and epidermal keratinocytes [28], they have also been detected in certain non-epithelial cells, including endothelial cells involved in angiogenesis [80]. The specific roles of cytokeratins during angiogenesis are still being investigated, and their gas plasma-induced upregulation may be linked to specific endothelial cell phenotypes, differentiation, interaction with the extracellular matrix (ECM), and potential regulatory mechanisms [80].

Vinculin expression is upregulated in response to pro-angiogenic factors, such as VEGF, necessary for endothelial cell migration and tube formation [81,82]. Vinculin localizes at the tips of angiogenic sprouts to regulate endothelial cell filopodia formation for sprouting angiogenesis [83]. We could demonstrate a strong switch of focal dynamics by strong vinculin localization on the tips of focal adhesion sites after gas plasma treatment. Vinculin also regulates the activity of other signaling molecules involved in angiogenesis, such as focal adhesion kinase (Fak) [31]. This was supported by increased Fak phosphorylation levels after gas plasma treatment. While syndecans are directly involved in angiogenesis [84], we determined their expression levels in wounds. Syndecans are a family of cell surface heparan sulfate proteoglycans (HSPGs) found in various cell types, including skin cells [30,85]. Syndecans contribute to skin development, homeostasis, and wound healing. In the latter, they are involved in cell adhesion, cell-matrix, and cell-cell interactions [86], signaling regulation (co-receptors for various growth factors and cytokines) [87,88], ECM organization and component interactions [89], and barrier function by regulating tight junction formation [90]. They participate in cell migration and growth factors and cytokine release to the wound site and participate in inflammatory responses during wound healing [91]. Targeting vinculin, Fak, or syndecans may have therapeutic potential in promoting or inhibiting angiogenesis in various physiological and pathological conditions, such as cardiovascular diseases.

Matrix metalloproteinases (MMPs) play a critical role in human diseases by degrading ECM components, such as collagen, fibronectin, and laminin, to allow endothelial cell migration and proliferation [92]. MMPs activate growth factors and cytokines, such as vascular endothelial growth factor (VEGF), which promotes endothelial cell proliferation and angiogenesis [93]. MMPs can be produced by various cells, including endothelial cells, smooth muscle cells, fibroblasts, and inflammatory cells [94]. However, excessive or uncontrolled MMP activity can lead to abnormal ECM degradation, impairing angiogenesis, and pathological conditions, such as impaired wound healing, chronic inflammation, and tumor progression [95]. Therefore, MMPs play a dual role in angiogenesis, promoting or inhibiting the process depending on their activity levels and the specific physiological or pathological context. Understanding the regulation of MMP activity and its role in angiogenesis is important for developing new therapeutic strategies for impaired angiogenesis conditions.

## 5. Conclusions

In our study, gas plasma exposure improved wound healing in diabetic animals, corroborating clinical evidence in humans. We linked this finding to several changes in these wounds, suggesting potential mechanisms of gas plasma-assisted diabetic wound healing, such as immediate oxygenation and blood flow changes in the tissue, a modulation of local inflammation, and alterations in matrix formation. Future studies in human wound tissue are needed to understand the clinical relevance of these findings.

## Data Availability

The underlying data of this manuscript are available from the corresponding authors upon reasonable request.

## Appendix A

**Table A1:** Murine gene-specific primers used in qPCR.

Gene Name	Gene ID	Primer Sequences (3′-5′)
nitric oxide synthase 2 (inducible)	*NOS2* (*iNOS*)	CCT GGT ACG GGC ATT GCT GCT CAT GCG GCC TCC TTT
cyclooxygenase 2	*COX2*	TGC CTG GTC TGA TGA TGT ATG CCA AGT AGT CGC ACA CTC TGT TGT GCT
platelet endothelial cell adhesion molecule 1	*CD31* (*PECAM1*)	CTG CCA GTC CGA AAA TGG AAC CTT CAT CCA CCG GGG CTA TC
keratinocyte growth factor	*KGF*	ACC TGA GGA TTG ACA AAC GAG G CCA CGG TCC TGA TTT CCA TGA
hypoxia-inducible factor 1A	*HIF1A*	GGG GAG GAC GAT GAA CAT CAA GGG TGG TTT CTT GTA CCC ACA
angiomotin	*AMOT*	CCG CCA GAA TACC CTT TCA AG GGT TCA GGC GAT GCT CAC TA
angiomotin like 1/2	*AMOTL1/2*	CCT TGC GAG CCT GTG CTT A AAG TCT GGG TAG AAG TAG GCG
vinculin	*VCL*	GCT TCAGTC AGACCC ATA CTC G AGG TAA GCA GTA GGT CAG ATG T
focal adhesion kinase	*FAK/PTK2*	GAG TAC GTC CCT ATG GTG AAG G CTC GAT CTC TCG ATG AGT GCT
syndecan 1	*SDC1*	AAC GGG CCT CAA CAG TCA G CCG TGC GGA TGA GAT GTG A
syndecan 4	*SDC4*	TTT GCC GTT TTC CTG ATC CTG TTG CCC AAG TCG TAA CTG CC
collagen 1A1	*COL1A1*	GCT CCT CTT AGG GGC CAC T ATT GGG GAC CCT TAG GCC AT
collagen 3A1	*COL3A1*	CTG TAA CAT GGA AAC TGG GGA AA CCA TAG CTG AAC TGA AAA CCA CC
decorin	*DCN*	GTC ATC TTC GAG TGG TGC AGT CAA GGT TGTGTCGGGTGGAAA
smooth muscle actin α	*α* *SMA*	CCC AGA CAT CAG GGA GTA ATG G TCT ATC GGA TAC TTC AGC GTC A
fibronectin 1	*FN1*	GCT CAG CAA ATC GTG CAG C CTA GGT AGG TCC GTT CCC ACT
matrix metalloproteinase 2	*MNMP2*	GAC ATA CAT CTT TGC AGG AGA CAA G TCT GCG ATG AGC TTA GGG AAA
matrix metalloproteinase 8	*MNMP8*	CTT ACC TCG GAT CGT AGT GGA CCC CAA CTA ACC CTC TTG AAG T
matrix metalloproteinase 9	*MNMP9*	CCT GGA ACT CAC ACG ACA TCT TC TGG AAA CTC ACA CGC CAG AA
matrix metalloproteinase 10	*MNMP10*	GAG CCA CTA GCC ATC CTG G CTG AGC AAG ATC CAT GCT TGG
matrix metalloproteinase 14	*MNMP14*	ACC CACACACAACGCTCAC GCC TGT CAC TTG TAA ACC ATA GA
inhibitor of matrix metalloproteinase 1	*TIMP1*	GCG TTT TGC AAT GCAGACG ATT CCC GGA ATC CAC CTCC
inhibitor of matrix metalloproteinase 2	*TIMP2*	GCC AAA GCA GTG AGC GAG AAG CAC ACT GCT GAA GAG GGG GC
glyceraldehyde 3-phosphate dehydrogenase	*GAPDH*	CAT GGC CTC CAA GGA GTA AG TGT GAG GGA GAT GCT CAG TG
ribosomal protein 13A	*RLP13A*	AGC CTA CCA GAA AGT TTG CTT AC GCT TCT TCT TCC GAT AGT GCA TC
β actin	*ACTB*	TTG CTG ACA GGA TGC AGA AG ACA TCT GCT GGA AGG TGG AC
